# Long-range PCR in next-generation sequencing: comparison of six enzymes and evaluation on the MiSeq sequencer

**DOI:** 10.1038/srep05737

**Published:** 2014-07-18

**Authors:** Haiying Jia, Yunfei Guo, Weiwei Zhao, Kai Wang

**Affiliations:** 1 The First Affiliated Hospital, Jinan University, Guangzhou, Guangdong 510632, China; 2 Zilkha Neurogenetic Institute, Keck School of Medicine, University of Southern California, Los Angeles, CA 90089, USA; 3 Department of Preventive Medicine, Keck School of Medicine, University of Southern California, Los Angeles, CA 90089, USA; 4 Kingmed Diagnostics, Guangzhou, Guangdong 510330, China; 5 Department of Psychiatry, Keck School of Medicine, University of Southern California, Los Angeles, CA 90089, USA

## Abstract

Long-range PCR remains a flexible, fast, efficient and cost-effective choice for sequencing candidate genomic regions in a small number of samples, especially when combined with next-generation sequencing (NGS) platforms. Several long-range DNA polymerases are advertised as being able to amplify up to 15 kb or longer genomic DNA. However, their real-world performance characteristics and their suitability for NGS remain unclear. We evaluated six long-range DNA polymerases (Invitrogen SequalPrep, Invitrogen AccuPrime, TaKaRa PrimeSTAR GXL, TaKaRa LA Taq Hot Start, KAPA Long Range HotStart and QIAGEN LongRange PCR Polymerase) to amplify three amplicons, with sizes of 12.9 kb, 9.7 kb, and 5.8 kb, respectively. Subsequently, we used the PrimeSTAR enzyme to amplify entire BRCA1 (83.2 kb) and BRCA2 (84.2 kb) genes from nine subjects and sequenced them on an Illumina MiSeq sequencer. We found that the TaKaRa PrimeSTAR GXL DNA polymerase can amplify almost all amplicons with different sizes and Tm values under identical PCR conditions. Other enzymes require alteration of PCR conditions to obtain optimal performance. From the MiSeq run, we identified multiple intronic and exonic single-nucleotide variations (SNVs), including one mutation (c.5946delT in *BRCA2*) in a positive control. Our study provided useful results for sequencing research focused on large genomic regions.

Since its inception, the polymerase chain reaction (PCR) has become one of the most indispensible tools in molecular biology to clone small DNA fragments^1^,^2^. However, traditionally PCR reactions were limited by the maximum size of amplified fragments. In 1992, Barnes^3^ developed new PCR conditions to allow for amplification of up to 5 kb. Long-range PCR increased the size of amplicons from 3–5 kb to over 30 kb by modifying the polymerases. These technical advances have brought the speed and simplicity of PCR to genomic mapping and sequencing, and have facilitated studies in molecular genetics^4^,^5^. When combined with sequencing, long-range PCR can achieve higher sensitivity and provide a faster and more cost effective tool for detecting genetic variations^6^,^7^.

Multiple long-range DNA polymerases are commercially available to amplify long genomic fragments. Some of them are advertised as being able to amplify up to 15 kb or longer genomic DNA and can work well for specific genomic regions under highly optimized conditions. However, little is known in literature (except manufacturer's flyer) on the advantage and disadvantages of each enzyme, and we are not sure about their real-world performance on randomly chosen amplicons. Since many next generation sequencing (NGS) experiments can benefit from long-range PCR, knowing the different characteristics of enzymes will have a significant impact on selecting enzymes and optimizing experimental conditions. Therefore, we compared six long-range DNA polymerases and attempted to amplify three amplicons with various sizes, to identify enzymes that have good performance with minimal requirements for condition optimization. Subsequently, we chose one enzyme to amplify the entire *BRCA1* and *BRCA2* genes (including introns and exons) for sequencing to further evaluate its performance for NGS.

A new generation of personal genome sequencers, such as the Illumina MiSeq and Ion Torrent PGM, are becoming popular in research and clinical settings. These sequencers have lower throughput and higher per-base-cost than Illumina HiSeq or Ion Proton, but their versatility and flexibility made them ideal for small labs where investigators prefer fast turn-around time. For example, the MiSeq sequencer allows assembly of small genomes or detection of variants in candidate regions with high accuracy, and the latest model can now generate 2 × 300 paired-end reads and up to 15 Gb of data in a single run. A previous study has successfully used long-range PCR to sequence *BRCA1* and *BRCA2* by Illumina Genome Analyzer II, and they have only tested one enzyme, the Invitrogen's SequalPrep^8^. In the current study, we selected Illumina MiSeq sequencer to determine if the combined use of long-range PCR and MiSeq can work well to identify exonic and intronic mutations in two important genes known to confer susceptibility to breast cancer.

## Methods

### Enzymes and Amplicons

We evaluated six commercially available long-range enzymes including SequalPrep polymerase (Invitrogen, Carlsbad, CA), AccuPrime Taq DNA Polymerase (Invitrogen, Carlsbad, CA), PrimeSTAR GXL polymerase (TaKaRa Bio, Shiga, Japan), LA Taq Hot Start Version Polymerase (TaKaRa Bio, Osaka, Japan), KAPA long Range HotStart DNA polymerase (KAPA Biosystems, Wobum, MA) and QIAGEN LongRange PCR Polymerase (Hilden, Germany). These enzymes were selected based on our knowledge at the time of the experiments, and based on Internet search. However, this is not a comprehensive list, and we acknowledge that other similar enzymes are also commercially available, such as New England Biolabs Phusion HF Polymerase and LongAmp Taq DNA Polymerase, Roche Expand Long Range DNA Polymerase, etc. Readers should not assume that the six enzymes used in the current study to be superior than those not included here.

Three amplicons were selected as the targets for comparing six long-range PCR enzymes, due to their variable lengths and variable Tm values for primers. The PCR primers of Brca1.1, 1.6 and 2.8 were synthesized by Integrated DNA technologies (Coralville, IA). The three PCR amplicons have sizes of 12.9 kb, 9.7 kb and 5.8 kb, and Tm values are 54°C, 63.3°C and 54.5°C, respectively (Table 1).

After comparing these six long-range PCR enzymes, we used PrimeSTAR to amplify all amplicons for the entire BRCA1/2 genes. Seventeen pairs of primers were synthesized by Integrated DNA technologies (Coralville, IA), where nine covered *BRCA1* and eight covered *BRCA2*, with sizes ranging from 5.8 kb to 13.6 kb (Table 1). Most of the primers were taken from Ozcelik et al^8^, and three pairs of primers were designed by Primer3^9^.

### Reaction mixture and PCR conditions

To evaluate the performance of different enzymes, we tested each enzyme to amplify DNA samples from de-identified human subjects. The study was reviewed and approved by the Institutional Review Board of the University of Southern California (#HS-14-00425). Each of six long-range PCR enzymes was used to amplify three amplicons using the same genomic DNA sample as the template. Because the amplification protocols of long-range PCR enzymes were different for different enzymes, all experiments were designed according to the reaction mixture and cycling conditions on the manual of the corresponding enzymes, and we also optimized PCR conditions according to the preliminary results for each enzyme. Reactions were performed using the Eppendorf Master Cycler (Hamburg, Germany). To measure the success of a long-range PCR amplification, the final PCR product was run on 0.8% agarose gel and visualized by staining with GelGreen Nucleic Acid Stain (Biotium, Hayward, CA). These amplicons were generated using the reaction mixture and PCR conditions listed in Table 2.

We further used the 2-step PCR condition of PrimeSTAR to amplify all amplicons in the BRCA1/2 genes. We found that the Brca 1.9 amplicon was difficult to amplify after re-designing multiple pairs of primers, possibly due to the presence of secondary structures during PCR amplification. After we added 0.4 μL dimethyl sulfoxide (DMSO) to 20 μL mixture reaction to interfere with the self-complementarity, the amplicon can be successfully amplified multiple times. All the other primers can be amplified using the standard 2-step protocol of PrimeSTAR.

### Library preparation and NGS for PCR amplicons

We purified all the amplicons using the Agencourt AMPure XP PCR Purification systems (Beckman Coulter, Pasadena, CA) and quantified the starting DNA library using the Qubit dsDNA BR Assay system (Invitrogen, Carlsbad, CA). The sequencing library construction was performed according to the Nextera XT sample preparation guide (Illumina, San Diego, CA) that uses transposome to fragment and simultaneously adds adapter and barcoding sequences.

The pooled and barcoded libraries were subsequently sequenced using the MiSeq sequencer with v2 kits, which generated 250-base paired-end sequence reads.

### Sequencing data analysis

The sequencing data analysis including quality control, mapping and variant calling was streamlined by SeqMule^10^, which consists of popular third party tools and then we used the wANNOVAR web server^11^ to annotate all the detected mutations.

First of all, sequencing data was evaluated with FastQC^12^. Short reads were aligned to reference genome (hg19) by BWA-MEM (version 0.7.4-r385)^13^ algorithm with default settings. Then we followed the GATK (Genome Analysis ToolKit) best practice to identify variants. GATK (version 2.8-1-g932cd3a)^14^ was used to realign reads and recalibrate base quality scores. Pre-processed BAM files were subjected to HaplotypeCaller of GATK for variant calling. The resulting SNPs were filtered by a set of filters including QD (quality by depth) <2.0, FS (Fisher strand bias test score) >60.0, MQ (root mean square of the mapping quality) <40.0, MappingQualityRankSum (mapping quality rank sum test score) <-12.5, ReadPosRankSum (read position rank sum test score) <−8.0. Indels were filtered by QD < 2.0, ReadPosRankSum < −20 and FS > 200. VQSR (Variant Quality Score Recalibration) method of GATK was not applicable due to limited number of variants. Then the wANNOVAR server was used to identify and annotate exonic and intronic variants, determine if the variants had been observed in public databases, and give predictions on whether non-synonymous variants were predicted to be deleterious based on multiple scoring systems.

## Results

### PCR results of six long-range PCR enzymes

**Figure 1:**
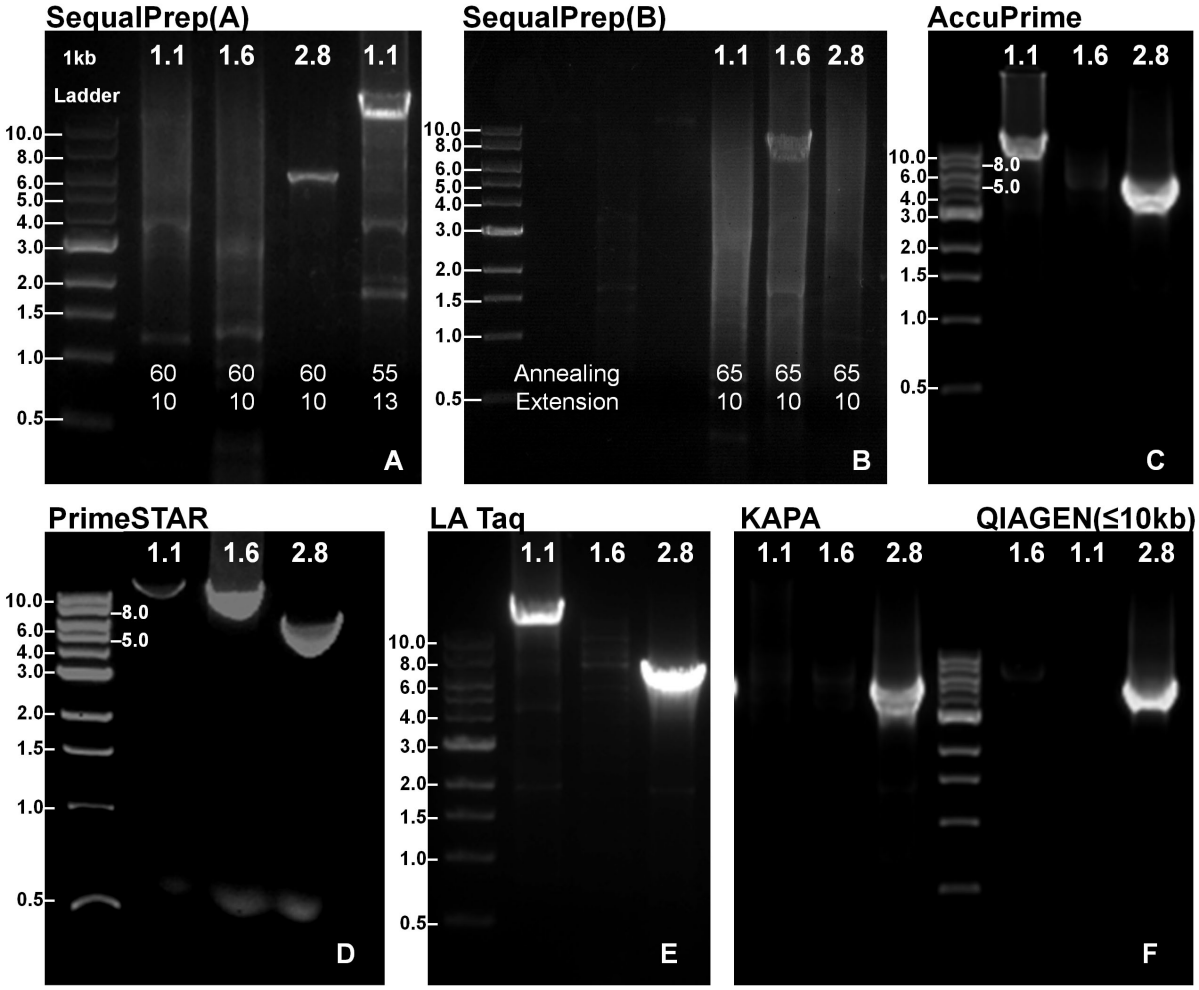
Gel electrophoresis of PCR products from the long-range PCR amplification by six enzymes. (A) and (B): SequalPrep (Three amplicons were amplified using amplicon-specific annealing temperature and extension time). (C): AccuPrime (Amplicons for Brca1.1 and 2.8 with similar Tm values were amplified). (D): PrimeSTAR (Three amplicons were amplified using a unified two-step PCR condition.). (E): LA Taq (Amplicons for Brca1.1 and 2.8 were amplified). (F): KAPA and QIAGEN (Only the amplicon for Brca2.8 was amplified).

We selected six long-range PCR enzymes for examination, each of which was advertised to be able to generate amplicons up to 15 kb or more (Table 3). We evaluated them on three amplicons with sizes of 12.9 kb, 9.7 kb, and 5.8 kb and Tm values of 54°C, 63.3°C and 54.5°C, respectively (Table 1). In summary, we found that both PrimeSTAR and SequalPrep Polymerases can amplify all three targets. AccuPrime and LA Taq can only amplify the 12.9 kb and 5.8 kb targets. KAPA and QIAGEN long Range polymerase can amplify the 5.8 kb target but not the two other larger ones (Figure 1).

The six enzymes require different PCR conditions to work properly (Table 2). The PrimeSTAR enzyme can use a unified two-step PCR condition to amplify all three targets, making experimental design and implementation for PCR much easier in real-world settings, as one single thermocycler can be used to amplify all targets simultaneously. However, the SequalPrep needs to use amplicon-specific annealing temperature and extension time, which for the three amplicons were 55°C and 13 minutes, 60°C and 10minutes, 65°C and 10 minutes, respectively. Both the LA Taq and AccuPrime can amplify 12.9 kb and 5.8 kb amplicons with similar Tm values using one PCR condition. The KAPA enzyme can amplify the 5.8 kb target, only after using the annealing temperature of 55°C with 13 minutes extension time, which represents the “longer targets cycle conditions” in the user manual. When we used the “very long range” reaction mixture and cycle conditions for the QIAGEN enzyme, none of the three amplicons can be amplified; however, after using the “long range” PCR conditions, the shortest target (5.8 kb) can be amplified (Table 4). All experiments were repeated at least twice to confirm these findings.

In addition to reaction time and tolerance to variation of cycling conditions, we were also interested in cost per reaction for these enzymes, for practical purposes of large-scale applications. Comparing the reaction time and price among these six enzymes, the PrimeSTAR polymerase stands out with 5 hours of PCR time and a cost of $0.4 per 20 μL reactions. Therefore, we chose the PrimeSTAR enzyme for long-range PCR for our NGS experiments below.

### Coverage and cost comparison of long-range PCR and custom capture arrays

To compare the target coverage of long-range PCR versus capture arrays, we used the Agilent SureDesign^15^ and NimbleGen SeqCap EZ Designs^16^ to design capture solutions for *BRCA1/2*. For Agilent solutions, we evaluated both SureSelect and HaloPlex. The designable coverage for exons is over 98% using all three designs. However, for exons and introns together, only HaloPlex design can achieve 96.6% coverage, yet SureSelect and SeqCap achieved coverage of 73.8% and 85.1%, respectively, suggesting reduced ability to cover intronic regions for these platforms. In comparison, the real-world performance of our long-range PCR method showed that it can get up to 100.00% coverage, even in a multiplex sequencing scenario where uneven sequencing depth exists across samples (Supplementary Table 1).

As with cost, both the SureSelect and HaloPlex might be four times as expensive as the long-range PCR method for library preparation, according to the quotes for capture probes and other reagents. However, this does not take into account labour costs or equipment costs, and some methods are more labour-intensive and error-prone than others. Furthermore, long-range PCR method may have higher specificity and uniformity than conventional capture method, and therefore requires lower sequencing coverage to obtain high-quality data^17^.

### Targeted Amplification of BRCA1 and BRCA2 Genomic Regions

To evaluate the PrimeSTAR polymerase in NGS settings, we amplified the entire genomic regions of BRCA1 (chr17:41196312-41279500, GRCh37/hg19) and BRCA2 (chr13:32889617-32973809, GRCh37/hg19). Initially we followed the primer sets reported by Ozcelik et al^8^, but a few amplicons cannot be amplified, despite multiple attempts to alter cycling conditions. Therefore, we re-designed some primer pairs, with the updated primers listed in Table 1.

**Figure 2:**
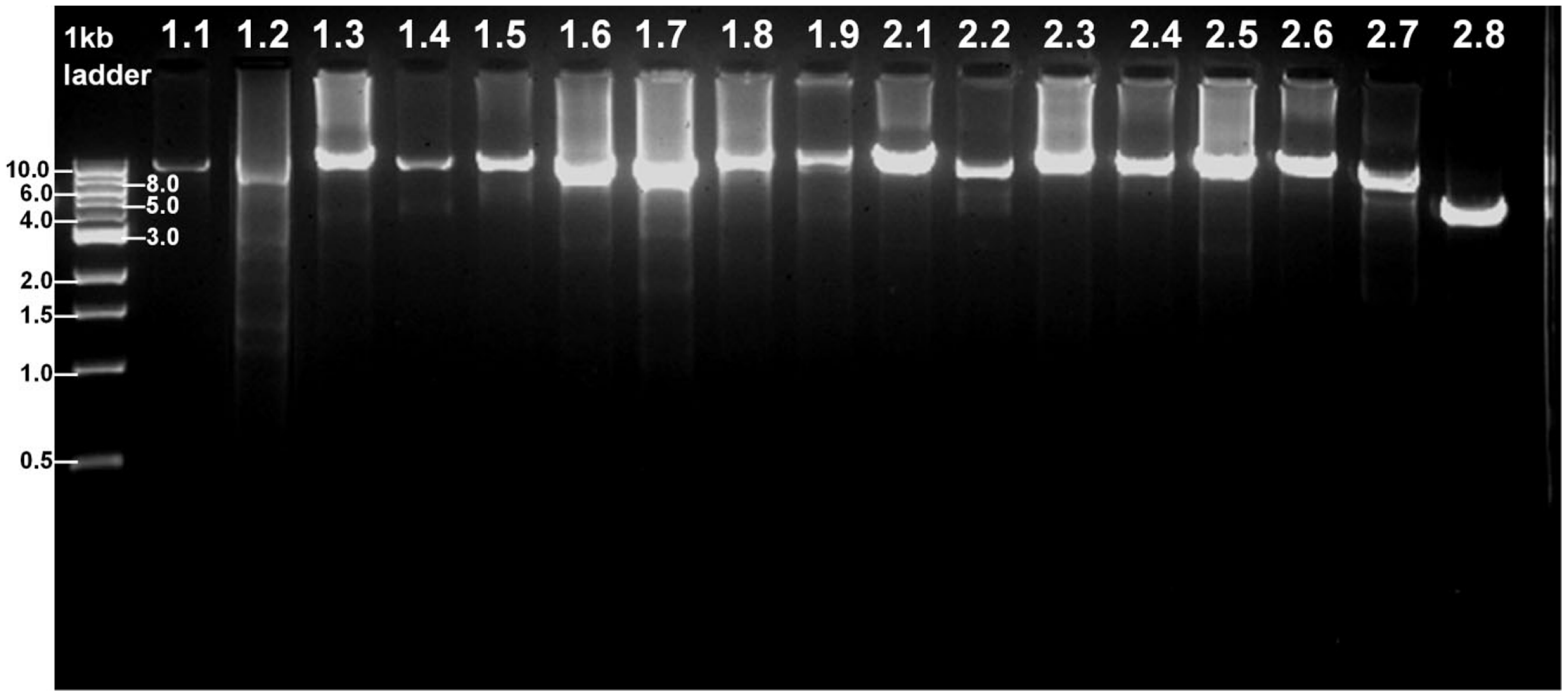
Successful results of 17 long-range PCR amplifications spanning the complete genomic region of BRCA1/2and their flanking sequences. Amplicons 1.1–1.9 cover BRCA1 and 2.1–2.8 cover BRCA2.

Nine DNA samples from peripheral blood of eight control subjects and one patient with hereditary breast cancer were used in our NGS experiments. Our goal is to evaluate if the experimental procedure can work consistently well among a group of samples and if a positive causal mutation can be identified reliably. For all samples, we were able to generate all the BRCA1/2 amplicons successfully, all of which display a single band with the expected size, without non-specific bands or smear (Figure 2).

### Sequencing the amplicons on MiSeq

We purified all amplicons, prepared sequencing libraries, and quantified the libraries using Qubit dsDNA BR Assay system (Invitrogen, Carlsbad, CA). Nine normalized libraries were pooled and sequenced together in one run on the Illumina MiSeq platform. Subsequently, we used BWA-MEM^13^ to align the sequencing reads, GATK software tool to call variants^14^, and the wANNOVAR web server^11^,^18^ to annotate variants detected from the sequencing data. On average, each sample had 4.6 million (range: 2.9–6.9) QC-passed reads, and 99.41% (range: 97.55% to 99.82%) of them can be properly aligned and paired. For each sample, 70.99% (range: 45.61% to 85.64%) of the reads can be mapped to the designed target region. The average coverage on the target regions was 2261X (range: 1285X to 3583X), and 93.75% (range:81.55% to 100.00%) of the target region had coverage of over 10 and 98% (range: 92.53% to 100%) of the target region was covered at least once (Supplementary Table 1).

**Figure 3:**
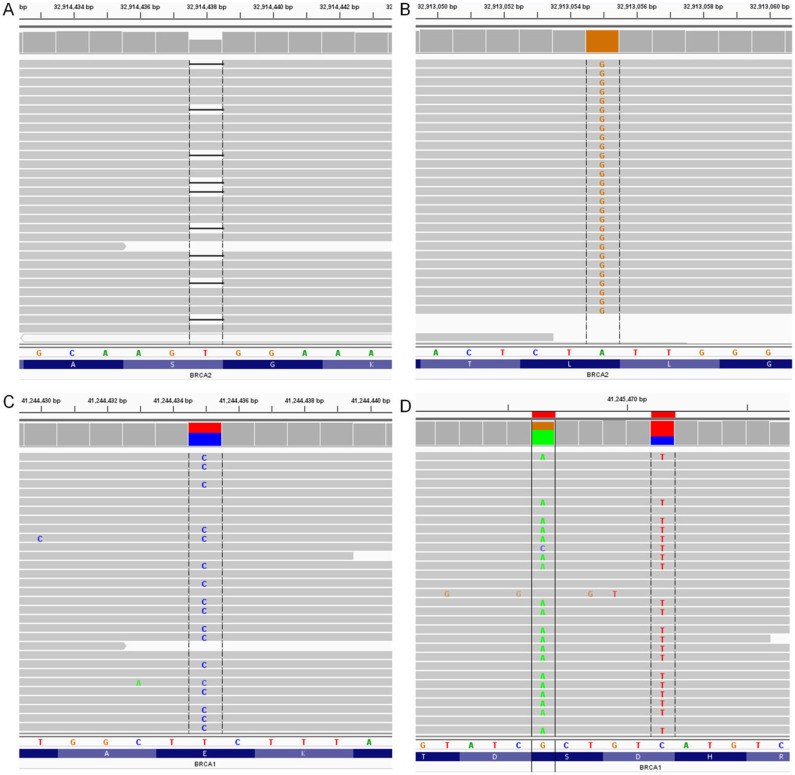
Visual validation of mutations. (A): A mutation (c.5946delT:p.S1982fs in BRCA2) from the sample with hereditary breast cancer; (B): A mutation (c.4563A > G in BRCA2, synonymous SNV) from all samples; (C): A mutation (c.2972A > G:p.E991G) from all samples; (D): Two mutations (c.1941C > T:p.S647S and c.1936G > A:p.D646N) in BRCA1 from sample 2, 7 and 8.

We examined the variant calls generated on these nine samples. On average, we identified 234 SNVs per sample, with the vast majority being non-coding variants. Based on variant annotation from the wANNOVAR web server, these nine samples carried 4, 8, 3, 7, 4, 2, 7, 7 and 6 non-synonymous SNVs, respectively. Additionally, we identified a nonframeshift deletion from one control subject and a frameshift deletion from the subject with hereditary breast cancer (Supplementary Table 2). This is a known disease causal mutation (c.5946delT in BRCA2) in the sample with hereditary breast cancer, and this mutation was verified by visualizing alignment on Integrative Genomics Viewer^19^ (Figure 3A). There are several mutations of unknown significance in other samples (Figure 3B–D). All non-synonymous SNVs found in our samples are listed in Supplementary Table 3.

**Figure 4:**
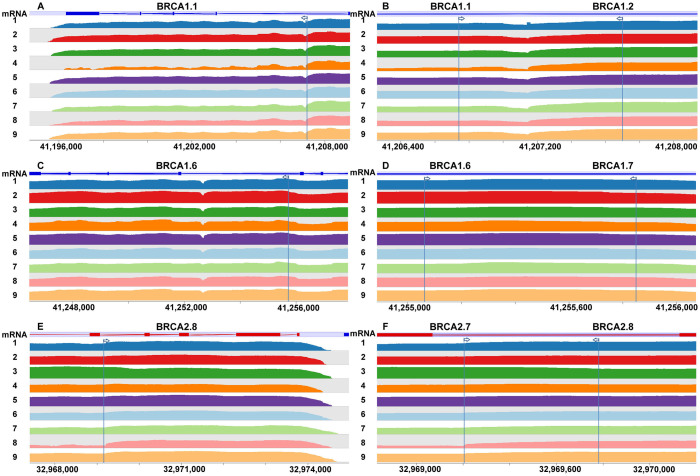
Visualization of sequencing read depth in SeqMonk for three amplicons previously used for comparing enzymes. Left column shows amplicons BRCA1.1, BRCA1.6 and BRCA2.8 and their boundaries. Right column shows junction regions. Arrows indicate boundaries. Coverage is log2-transformed. The coverage plot demonstrates that significant variations of read depth may still exist within and between amplicons from long-range PCR. Loss of coverage may occur at junction of two amplicons (B), but can be recovered by larger overlapping of two amplicons (D, F).

One potential advantage of long-range PCR-based NGS might be that the sequence coverage is more likely to be even, given that the same amount of starting DNA material is available for all fragments from the same amplicon. We used Wiggle plot in SeqMonk^20^ to view sequencing read depth of the three amplicons in BRCA1 and BRCA2 that were used in the comparative analysis of six enzymes (Figure 4). The coverage plot demonstrated that significant variations of read depth may still exist even for regions in the same amplicon from long-range PCR. Different amplicons (for example, BRCA1.1 and BRCA1.2) may also have different coverage, which may be improved by better sample normalization during library preparation. Additionally, we found that the relative sequencing depth for the same region tends to correlate across samples, suggesting that coverage correlates with certain sequence features such as GC content, repetitive sequence and Nextera restriction enzyme sites. At the rims of amplicons, coverage tends to be lower than the neighbouring region (e.g. BRCA1.1-BRCA1.2 junction). This loss of coverage can be recovered by larger overlapping between two amplicons (Figure 4D, 4F). Based on our observation, 1 kb overlapping of two amplicons seems to be sufficient (Figure 4F). In summary, long-range PCR is not immune to uneven sequence coverage typically observed in NGS experiments for capture arrays.

## Discussion

Long-range PCR has been commonly used to prepare specific high-molecular-weight DNA fragments for a variety of applications, including cloning, genome mapping and sequencing, and contig construction^21^. Generally speaking, to successfully amplify all amplicons in an experiment, one needs to change the annealing temperature and extension time, which are specific to each amplicon because the primers may have very different Tm values. In our experiment, we found that the TaKaRa PrimeSTAR GXL DNA Polymerase can amplify all amplicons of BRCA1/2 without altering experimental conditions, which we believe is an key advantage of using this enzyme when resources such as thermocycler is a limiting factor in research and clinical settings.

In addition to long-range PCR, a variety of other methods, such as solution-based capture, microarray-based capture, molecular inversion probes (MIPs) and multiplex PCR, have been used in target enrichment applications. Target enrichment is a highly effective way of reducing costs and saving time when only specific genomic regions (such as all exons in a gene, or a genomic region spanning a few GWAS loci) are of interest. Approaches based on capture, such as solution-based capture and microarray-based capture, achieve high-performance and have advantages for medium to large target regions (10–50 Mb)^22^. However, the microarray-based methods, such as Agilent SureSelect and HaloPlex, require large amounts of input DNA to be successful as well as expensive hardware working with microarray slides^17^,^23^; solution-based capture, such as NimbleGen SeqCap, is less extensively used because of performance issues^24^ but the solution-based capture techniques are constantly improving. Generally speaking, GC-rich segments were not well-represented in capture samples. This may be attributed to sequencing bias, as well as difficulty in capture for high GC template^23^. This is less a concern for the long-range PCR that “capture” large regions at once, especially for specific enzymes (such as PrimeSTAR GXL and QIAGEN LongRange PCR polymerase) that were optimized for amplifying GC-rich segments. MIPs are generally believed to be superior in terms of specificity, but far less amenable to multiple sample co-processing in a single reaction. Moreover, its design has to consider the uniqueness of each target region fragment and the most suitable hybridization conditions^22^. Long-range PCR has its unique niche, in that it does not require customized design by commercial vendors, and can be afforded by small laboratories when a small number of samples and continuous regions (such as full gene region including introns) are of interest.

Although mutations in the coding regions of BRCA1/2 have been heavily studied in previous genetic studies, potentially deleterious alterations may also reside in the less studied non-coding intronic sequences. For example, an insertion/deletion mutation in intron 24 (3′ UTR) of BRCA1 gene was found in one of the families with five breast cancer patients^25^. Additionally, a novel intronic mutation (IVS7 + 34_47delTTCTTTTCTTTTTT) and two unclassified intronic variations (IVS7 + 34_47delAAGAAAAGAAAAAA in the antisense strand and IVS7 + 50_63delTTCTTTTTTTTTTT in the sense strand) in BRCA1 were identified in a Thai family with a history of breast cancer^26^. Olgaet et al^27^ reported that an intronic mutation (c.6937 + 594T > G) can activate a cryptic exon in BRCA2 that disrupts the coding sequence in breast cancer families. For these regions, to gain a more comprehensive understanding of the genotype-phenotype relationships on BRCA1/2, it is necessary to examine both intronic and exonic regions.

In this study, we compared 6 long-range DNA polymerases for amplification of three amplicons, with sizes of 12.9 kb, 9.7 kb, and 5.8 kb, respectively, and found that the TaKaRa PrimeSTAR GXL DNA polymerase can amplify almost all amplicons with different sizes and Tm values under identical PCR conditions. We demonstrated that real-world performance for enzymes vary greatly between manufacturers, despite advertised performance characteristics, and how to couple long-range PCR with MiSeq sequencer which results in much faster turnaround time than previously possible. Overall, this report provides a practical guide on how to use long-range PCR to perform NGS on large genomic regions, especially when the entire gene regions including introns are of interest.

## Author Contributions

H.J. performed the experiments and analyzed the data. Y.G. offered technical support for data analysis and SeqMule. W.Z. provided materials and advised on the design and implementation of the study. K.W. conceived and supervised the study.

## Supplementary Material

Supplementary Information Supplementary Dataset 123

## Figures and Tables

**Table 1:** Primer Sequences for the BRCA1 and BRCA2 Genomic Region

Primer ID	Genomic sequence (GRCh37/hg19)	Primer Sequence	Tm (°C)	Length of Amplicon (bp)
**Brca1.1^#^**	Chr17: 41194339-41207207	5′-ACCCCAACATTGATTCCTTTC-3′	53.2	12896
		5′-CACAGGGAGAAAGTCTGCAAG-3′	55.7	
Brca1.2	Chr17: 41207139-41215625	5′-GGGAGCTGAGAAAGCAGCCAGC-3′	62.9	8487
		5′-TCGGCAGGAATCCATGTGCAGC-3′	62.4	
Brca1.3	Chr17: 41215424-41229078	5′-AGCAGAAGAACGTGCTCTTTTCACGG-3′	61.2	13655
		5′-ACAGTCTTCAATGTGGAGGCAGTAGGG-3′	61.8	
Brca1.4	Chr17: 41227538-41239085	5′-CTGGATTGAAGATGGGTGAGA-3′	54.1	11548
		5′-TTTCCTGTACCTTGCCAACAC-3′	55.5	
Brca1.5	Chr17: 41238861-41251840	5′-GGCAATCCTGAAGAAGTGGA-3′	54.8	12980
		5′-ACAAAGCAGCGGATACAACC-3′	55.8	
**Brca1.6^#^**	Chr17: 41246132-41255853	5′-GGGGAGGCTTGCCTTCTTCCG-3′	62.9	9722
		5′-CTGTGCCCGGCCGGTAAAACC-3′	63.6	
Brca1.7	Chr17: 41255072-41264003	5′-GCCATGGCACCCAGCTGAAGTA-3′	62.1	8932
		5′-CTGGGAGCGATACCCCCATGCT-3′	63.4	
Brca1.8	Chr17: 41258624-41271477	5′-GCCATGAAAAGATAATCTCACAACTGC-3′	56.6	12854
		5′-GGTGGCTCTGCTTATATACACAACTGG-3′	59.0	
Brca1.9	Chr17: 41270116-41281112	5′-GAAAGGTTTCACTGAGGTGAGACTA-3′	56.2	10997
		5′-ACAAGTTAGCTTTTCCTCCACATC-3′	55.3	
Brca2.1	Chr13: 32888055-32900396	5′-CTCCCCCACAAAAAGGGGACAAAGC-3′	62.3	12342
		5′-ACAAACTCCCACATACCACTGGGGG-3′	62.6	
Brca2.2	Chr13: 32900267-32911248	5′-CACCACAAAGAGATAAGTCAGGTAT-3′	54.3	10982
		5′-TCGTTTACACAAGTCAAGTCTG-3′	52.8	
Brca2.3	Chr13:32910302-32923088	5′-GCCACTGTGCCCAAACACTACC-3′	60.9	12787
		5′-TGTGCCTGGCCTCAATTCACCA-3′	61.6	
Brca2.4	Chr13:32922508-32935813	5′-TGACCCACAGTAAGGCACATC-3′	57.0	13306
		5′-GCCCTCTTCTACCATTTGTGC-3′	56.1	
Brca2.5	Chr13:32933399-32945178	5′-GGCCTTATGGTAGATTCCTCCCCCG-3′	62.5	11780
		5′-TGGGCCTCCACATATTTTGCTGCTT-3′	61.3	
Brca2.6	Chr13:32945170-32958766	5′-GGAGGCCCAACAAAAGAGAC-3′	56.1	13597
		5′-GTGGTTTAGCCGGACTCCTC-3′	57.6	
Brca2.7	Chr13: 32958373-32969763	5′-GGCAACTGTACAGGCAGACA-3′	57.5	11391
		5′-GTCTGGGTTCTGCATTCGAT-3′	55.0	
**Brca2.8^#^**	Chr13: 32969199-32974976	5′-TTAATTGCCCATGAACCTCAG-3′	53.0	5778
		5′-TATTGACTTGTATTGTGTTCGCTGT-3′	54.9	

^#^The three amplicons selected for comparing six long-range PCR enzymes.

**Table 2:** The protocol of the long-range PCR reactions for different enzymes

Enzymes	The composition of reaction mixture	PCR conditions
SequalPrep	70 ng template DNA, 2 *μ*L of a 2.5 *μ*mol/L primers, 2 *μ*L 10× Reaction Buffer, 1 *μ*L PCR enhancer, 0.4 *μ*L DMSO, 0.36 *μ*L Polymerase and sterilized distilled water up to 20 *μ*L	94°C 2 minutes
		10 cycles
		94° 10 seconds
		Tm-5° (55, 60 or 65°C) 30 seconds
		68°C 10/13 minutes
		25 cycles
		94°C 10 seconds
		Tm-5° (55, 60 or 65°C) 30 seconds
		68°C 10/13 minutes plus 20 seconds
		72°C 5 minutes
		hold at 4°C
AccuPrime	70 ng template DNA, 3.2 *μ*L of 2.5 *μ*mol/L primers, 2 *μ*L 10× Buffer II, 0.1 *μ*L polymerase, and distilled water to 20 *μ*L.	94°C 30 seconds
		30 cycles
		94°C 30 seconds
		60°C 20 seconds
		68°C 13 minutes
		Hold at 4°C
PrimeSTAR	35 ng template DNA, 3.2 *μ*L of 2.5 *μ*mol/L primers, 4 *μ*L 5× buffer, 1.6 *μ*L dNTP, 0.4 *μ*L Polymerase, and distilled water to 20 *μ*L.	30 cycles
		98°C 10 seconds
		68°C 10 minutes
		Hold at 4°C
LA Taq	35 ng template DNA, 4 *μ*L of 2.5 *μ*mol/L primers, 2 *μ*L 10× Buffer II, 3.2 *μ*L dNTP, 0.2 *μ*L polymerase, and distilled water to 20 *μ*L.	94°C 1 minute
		30 cycles
		98°C 10 seconds
		68°C 12 minutes
		72°C 10 minutes
		Hold at 4°C
KAPA 5–18 kb	35 ng template DNA, 8 *μ*L of 2.5 *μ*mol/L primers, 2 *μ*L 5× Buffer, 0.6 *μ*L dNTP, 1.4 *μ*L 25 mM Mgcl_2_, 0.2 *μ*L polymerase(2.5 U/ *μ*L), and distilled water to 20 *μ*L	94°C 30 seconds
		35 cycles
		94°C 20 seconds
		60°C 15 seconds
		68°C 12 minutes
		72°C 10 minutes
		Hold at 4°C
		QIAGEN
(0.1–10 kb)	35 ng template DNA, 6.4 *μ*L of 2.5 *μ*mol/L primers, 2 *μ*L Buffer, 1 *μ*L dNTP, 0.2 *μ*L enzyme, and distilled water to 20 *μ*L.	94°C 3 minutes
		35 cycles
		93°C 15 seconds
		62°C 30 seconds
		68°C 10 minutes
		Hold at 4°C
(>10 kb)	70 ng template DNA, 6.4 *μ*L of 2.5 *μ*mol/L primers, 2 *μ*L Buffer, 1 *μ*L dNTP, 0.2 *μ*L enzyme, and distilled water to 20 *μ*L.	94°C 3 minutes
		10 cycles
		93°C 15 seconds
		62°C 30 seconds
		68°C 13 minutes
		28 cycles
		93°C 15 seconds
		62°C 30 seconds
		68°C 13 minutes plus 20 seconds
		Hold at 4°C

**Table 3:** A list of enzymes for long-range PCR reaction.

Enzyme ID	Product	Company	Amplicon size (kb)	high-fidelity	Hot-start	For GC-rich templates	Proofreading activity	Reaction time (13 kb)	Price per 20 μL reaction
SequalPrep	SequalPrep Long PCR Polymerase	Invitrogen	15	Yes	Yes	-	-	~7 h	$1.6
AccuPrime	AccuPrime Taq DNA Polymerase	Invitrogen	20	Yes	Yes	-	Yes	~7 h	$0.6
PrimeSTAR	PrimeSTAR GXL DNA Polymerase	TaKaRa	>30	Yes	Yes	Yes	-	~5 h	$0.4
LA Taq	LA Taq Hot Start Version	TaKaRa	>15	-	Yes	-	-	~6 h	$1.4
KAPA	long Range HotStart DNA polymerase	KAPA	20	Yes	Yes	-	Yes	~7 h	$0.5
QIAGEN	LongRange PCR polymerase	QIAGEN	40	Yes	-	Yes	Yes	~8.5 h	$1.0
-	ACCUZYME DNA Polymerase	BIOLINE	5	Yes	-	-	Yes	-	-
-	KOD Hot Start DNA Polymerase	Novagen	12	Yes	Yes	Yes	Yes	-	-

**Table 4:** Primer-specific PCR conditions and results

	SequalPrep																			QIAGEN					
	A				B			AccuPrime			PrimeSTAR			LA Taq			KAPA			0.1-10 kb			>10 kb		
**Sample**	1	2	3	1	1	2	3	1	2	3	1	2	3	1	2	3	1	2	3	2	1	3	1	2	3
**Annealing(**°**C)**	60	60	60	55	65				60		-				-			60			62			62	
**Extension(min)**	10	10	10	13	10				13			2-step			12			12			10			13	
**Results**	-	-	+	+	-	+	-	+	-	+	+	+	+	+	-	+	-	-	+	-	-	+	-	-	-
